# Impact of Environmental Enrichment on Perineuronal Nets in the Prefrontal Cortex following Early and Late Abstinence from Sucrose Self-Administration in Rats

**DOI:** 10.1371/journal.pone.0168256

**Published:** 2016-12-15

**Authors:** Megan Slaker, Jesse Barnes, Barbara A. Sorg, Jeffrey W. Grimm

**Affiliations:** 1 Department of Integrative Physiology and Neuroscience and Translational Addiction Research Center, Washington State University, Vancouver, Washington, United States of America; 2 Department of Integrative Physiology and Neuroscience, Washington State University, Pullman, Washington, United States of America; 3 Department of Psychology and Behavioral Neuroscience Program, Western Washington University, Bellingham, Washington, United States of America; Radboud University Medical Centre, NETHERLANDS

## Abstract

Perineuronal nets (PNNs) are aggregates of extracellular matrix that form structures surrounding a subset of GABAergic interneurons. The staining intensity of PNNs appears to be related to plasticity. Environmental enrichment (EE) influences plasticity during adulthood: EE decreases the rewarding effects of drugs of abuse and diminishes both drug- and sucrose-seeking behavior. We determined the impact of EE on PNN intensity in the medial prefrontal cortex (mPFC) in rats trained to self-administer sucrose. We examined the number and intensity of PNNs within the prelimbic (PL), infralimbic (IL), and orbitofrontal (OF) regions of the mPFC of adult Long-Evans rats that were trained for sucrose self-administration followed by acute or chronic EE during abstinence and a cue-induced reinstatement test. Rats exposed to EE prior to a cue-induced reinstatement of sucrose seeking had an increase in PNN staining compared with rats in standard housing. Conversely, naïve rats given 1 day of EE had a decrease in PNN intensity in the PL, no change in the IL, and an increase in the OF. Our findings demonstrate that EE increases PNN intensity in the mPFC after sucrose training, suggesting that training enhances the ability of EE to increase PNN intensity. We further demonstrate an interaction between time of abstinence, duration of EE exposure, and cue-induced reinstatement. Our results suggest that increased PNN intensity after EE may alter the excitatory/inhibitory balance of mPFC neurons such that rats are less responsive to a sucrose cue.

## 1. Introduction

The environment has a profound impact on the brain and behavior. Exposure to an enriched environment increases learning and memory processes, dendritic branching, and synapse formation in several areas of the brain (for review see [1]). This environmental enrichment (EE) also decreases the effects of chronic stress [2, 3]. Additionally, EE prevents and protects against the effects of drugs of abuse. Exposure to EE either prior to or following drug-seeking behaviors (sensitization, conditioned place preference (CPP), and self-administration), decreases the rewarding effects of cocaine and methamphetamine and diminishes drug-seeking behavior [4–9]. Exposure to EE also decreases cue-induced reinstatement of *sucrose* seeking [10–12]. This decrease in behavior coincides with decreased protein expression of the immediately early gene, c-Fos, within areas of the brain important for reward-related behaviors, including the prefrontal cortex (PFC), amygdala, and nucleus accumbens [12–14]. While these studies demonstrate that EE decreases reward-seeking behaviors and point to several brain regions that may mediate this effect, the underlying mechanisms remain unknown.

An area of recent intense interest is focused on the role of the extracellular matrix (ECM) on the plasticity related to reward-seeking behavior [15]. In the present study, we determined the impact of EE and sucrose-seeking behavior on perineuronal nets (PNNs) within several regions of the PFC. PNNs are aggregations of ECM molecules that form net-like structures surrounding the cell body and proximal dendrites of mainly a subset of fast-spiking, GABAergic interneurons within the central nervous system [16, 17]. PNNs appear during development in an experience-dependent manner and are important for plasticity during critical periods and during drug-associated learning and memory [18–21]. Within the visual cortex, the appearance of PNNs coincides with the closure of the critical period for ocular dominance plasticity, and removal of PNNs with the bacterial enzyme, chondroitinase-ABC, within the visual cortex re-opens the critical period window and allows for remodeling of ocular dominance columns in adults [19]. Additionally, removal of PNNs with chondroitinase-ABC from the amygdala or prelimbic (PL) region of the PFC alters drug-induced plasticity, resulting in decreased drug-seeking behavior, either by enhancing extinction training [21] or by impairing reconsolidation [20].

Chronic EE is also capable of modulating PNNs. Exposing an adult animal with visual impairments to chronic EE reinstates visual acuity and decreases the intensity of PNN staining within the visual cortex [22, 23]. Additionally, exposure to EE decreases the number and intensity of PNN staining within the somatosensory cortex, motor cortex, and cerebellum [24, 25]. These studies suggest that PNNs are modulated by experience even during adulthood. Since PNNs have been implicated in plasticity associated with both EE and reward-seeking behaviors, we sought to determine the impact of EE on PNN staining within several regions of the PFC important for reward-related behaviors [26, 27]. Here, we assessed staining intensity and number of PNNs within the PL, infralimbic (IL), and orbitofrontal (OF) regions of the PFC in rats trained for sucrose self-administration.

## 2. Materials and Methods

### 2.1 Subjects: Sucrose Self-administration and Acute and Chronic EE

Tissue for PNN analyses was derived from 33 Long-Evans rats (Simonson) weighing 452 ± 6.6 g (mean ± SEM) at the start of the study. Rats were housed in a temperature- and humidity-controlled room under a reverse light/dark cycle (lights off at 07:00). These rats represent a subset of animals used in a study examining brain regional c-Fos levels following acute or chronic EE and the incubation of sucrose craving. Detailed methodological information is found in the publication of that study, Grimm et al. [12]. All procedures with these animals followed the guidelines outlined in the “PHS Policy on Humane Care and Use of Laboratory Animals (2002)” and were approved by the Western Washington University Institutional Animal Care and Use Committee.

### 2.2 Sucrose Self-administration and Acute or Chronic EE

**Fig 1:**
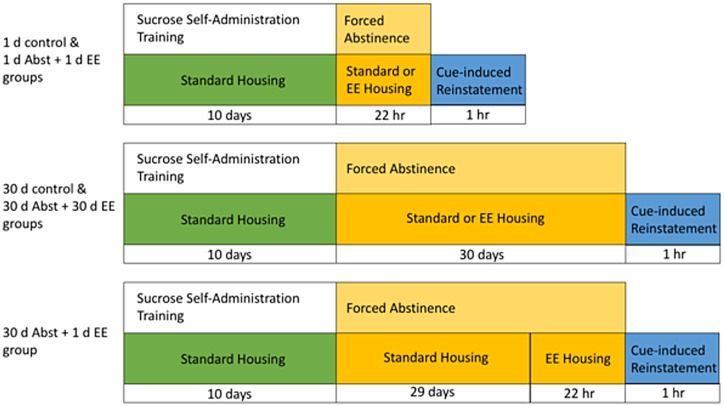
Experimental timeline of sucrose self-administration, abstinence, and cue-induced reinstatement for each group.

Detailed information regarding the operant procedures is provided in Grimm et al. [12]. A summary of the experimental timeline and groups is shown in Fig 1. Briefly, rats lever-pressed (active lever) for 10% sucrose (0.2 mL) in 10 daily 2-hr training sessions on a fixed-ratio 1 schedule of reinforcement with a 40 sec “time-out” period following each sucrose delivery in which active lever presses were recorded but provided no sucrose reward. A 5-sec duration tone and white stimulus light accompanied each sucrose delivery. Presses on a separate lever (inactive lever) were recorded but had no consequences. Locomotor activity was recorded during all sessions using infrared photobeam breaks.

Following the tenth training session, rats were randomly assigned to a treatment condition, consisting of a cross between duration of forced abstinence (Abst) and type of housing condition (standard or EE), based on average active lever presses during training. The Abst period was either from the end of the tenth training session to a testing session the next morning (~22 hr; “1 d Abst”) or to a testing session 30 days later (“30 d Abst”).

The EE housing was a large, 4-level wire-mesh environment (91 X 51 X 102 cm; Quality Cage Company, Portland, OR) with novel toys replenished each M, W, F. Three rats were co-housed in EE. 1 d EE groups were created so that rats experienced EE from the end of the tenth day of training (1 d Abst + 1 d EE) or the 29^th^ day of Abst (30 d Abst + 1 d EE) until testing the next morning (~17 hr). The chronic EE condition (30 d Abst + 30 d EE) was exposure to EE from the end of the tenth day of training until testing on day 30 of Abst. All control conditions were simply allowing rats to remain single-housed.

A reinstatement test session was identical to a training session except that sucrose was not available and the session length was 1 hr. All subjects received a test session. Immediately following testing, subjects were deeply anesthetized with pentobarbital (Soccumb; Butler-Schein, Dublin, OH) and trans-cardially perfused with ice-cold 4% paraformaldehyde (PFA; Electron Microscopy Sciences, Hatfield, PA). Brains were extracted and placed in 4% PFA for ~24 hr, then a 20% sucrose solution (MP Biomedicals, Santa Ana, CA) for 24 hr, and finally into 30% sucrose for 24 hr or until the brain sank, all at 4°C. Brains were then frozen in dry ice powder, wrapped in foil, and stored at -70°C until sectioned.

### 2.3 Subjects: Novel Acute Environmental Enrichment

A total of 12 male Long-Evans rats (Simonsen Laboratories, Gilroy, CA) were used for this experiment. Rats weighed 434 ± 20 g at the time of EE. Rats were housed in a temperature- and humidity-controlled room with a 12 hr light/dark cycle (lights off at 7:00 a.m.). Experiments were conducted according to the National Institutes of Health Guide for the Care and Use of Laboratory Animals and the Washington State University Institutional Animal Care and Use Committee. Rats were singly housed for at least 7 days prior to the start of the experiment. Food and water were available *ad libitum*.

### 2.4 Novel Acute Environmental Enrichment

Acute EE lasted for 22 hr. Rats were pair housed in a large (20 cm x 35.5 cm x 48.25 cm) plastic cage with two ceramic bowls and two plastic toys (one cube-shaped, one cylinder-shaped). Extra food and water were provided to ensure that both animals had full access. Control rats remained singly housed, but at the time the rats began EE, control rats were moved to a new cage with fresh bedding in a different location in the vivarium. Following 22 hr of acute EE, animals were perfused intracardially with ice-cold 4% PFA dissolved in PBS. Brains were stored in 4% PFA for 24 hr, moved to 20% sucrose for 24 hr, and then flash frozen and stored until use.

### 2.5 WFA staining

40 μm coronal sections of the PFC (+3.2 from bregma) were obtained using a freezing microtome (novel acute EE) or using a Leica (Buffalo Grove, IL) CM1950 cryostat (sucrose self-administration and EE). The sucrose study slices were placed into pH 7.4 cryoprotectant consisting of 20% glycerol and 2% DMSO (both from Fisher, Pittsburgh, PA) in 6-well plates (Corning, Tewksbury, MA), shipped on dry ice, and stored at -80°C until staining. Free-floating sections were washed three times in 1x-PBS for 5 min each and then quenched for 30 min in 50% ethanol. Following an additional set of three, 5 min rinses in 1x-PBS, sections were incubated overnight (~20 hr) in 1:500 fluorescein-conjugated *Wisteria floribunda* agglutinin (WFA, Vector Laboratories) in 2% goat serum (Vector Laboratories) in PBS. The next morning, sections were washed and mounted on Frost plus slides in diluted PBS with Triton in deionized water (6:1:41). Slides were cover slipped with ProLong Gold (Life Technologies) and stored flat and dark until time of imaging.

### 2.6 Imaging and Analysis

Images of the PL, IL, and OF regions of the PFC were taken using a Leica SP8 laser scanning confocal microscope with Leica Application Suite. An HCX PL apo CS, dry, 20X objective with 0.70 numerical aperture was used for all images. WFA-bound fluorescein was excited using a 488 laser, and a photomultiplier tube detected emission photons within the range of 495–545 nm. Images were taken through a z-plane (9 μm) at the center of the tissue containing 25 stacks within each region. Gain, offset, laser intensity, zoom, and pinhole were kept constant for all images. Sequences of the raw images within the z-stack were exported and projected into a sum slices image using ImageJ software (NIH).

Quantification of WFA intensity from one bilateral PFC section (+3.7 from bregma) was conducted as described in Slaker et al. [28]. Briefly, background subtraction from each projection image was conducted by first using the Rolling Ball Radius function to remove smooth continuous background and then manually determining two standard deviations above the mean within a region of the image containing no visible PNNs to separate PNN staining from general extracellular matrix staining. Each visible PNN (surrounding at least 2/3 of the cell body) in the image was assigned as a region of interest, including the cell body and proximal dendrites. The average intensity above background from each region of interest was calculated. These raw intensity values were normalized to the 1 d control group average to compare across staining groups.

### 2.7 Statistics

All statistical tests were conducted using Prism6 (Graph Pad, Inc.) or SPSS (IBM, Inc.) software. Behavioral data were compared among groups using a two-way (Day of abstinence X Housing condition) ANOVA using the Type 4 Sum of Squares model. As PNN intensity data were generally not normally distributed, non-parametric testes were used to identify group differences. The Kolmogorov-Smirnov nonparametric test was used to compare intensity of PNNs between two conditions and Kruskal-Wallis nonparametric, one-way ANOVA tests were used to compare the intensity of PNNs among treatment conditions, with Dunn’s multiple comparisons tests to further explore significance from the Kruskal-Wallis test. Numbers of PNNs were normally distributed allowing use of two-tailed, Student’s t-tests and one-way ANOVAs to compare the number of PNNs among treatment conditions.

## 3. Results

### 3.1 Cue-induced reinstatement of sucrose self-administration increased over abstinence and decreased following EE

Table 1 shows a summary of the behavioral data for the sucrose self-administration and cue-induced reinstatement animals during 10 d of training followed by a cue-induced reinstatement test. A summary of the experimental timeline and groups is shown in Fig 1. No significant differences were observed between groups prior to housing and/or abstinence manipulations. Body weights also did not differ between Controls and EE subjects prior to or following housing manipulations and cue-reactivity testing (data not shown). Two-way ANOVA of Testing behavior revealed both incubation of sucrose craving and EE-induced decreases in sucrose seeking. For Active lever presses, there was a nearly significant effect of Day (F(1,28) = 4.1, p = 0.052), and a significant effect of Housing (F(2,28) = 10.4, p < 0.001). For Infusions (cue-deliveries contingent on active lever responses) there was a significant effect of Day (F(1,28) = 4.4, p < 0.05), and a significant effect of Housing (F(2,28) = 11.0, p < 0.001). There were no significant effects or interactions for Inactive lever presses. For Photobeam Breaks there was a significant effect of Housing (F(2,28) = 11.4, p < 0.001).

**Table 1:** Summary of behavioral data from sucrose self-administration training (average of 10, 2-hr training sessions) and cue-induced reinstatement (1 hr session).

		Training (Average ± SEM)				Cue-Induced Reinstatement (Average ± SEM)			
Group	n	Active Lever Presses	Infusions	Inactive Lever Presses	Photobeam Breaks	Active Lever Presses*+	Infusions*+	Inactive Lever Presses	Photobeam Breaks+
Control—1 d Abst	6	147.8± 23.0	88.8 ± 11.3	15.8 ± 7.5	1912.4 ± 310.2	50.2 ± 6.8	21.0 ± 2.6	4.1 ± 2.6	1137.2 ± 258.3
Control—30 d Abst	4	128.8 ± 11.9	81.1 ± 8.7	4.2 ± 2.1	2070.7 ± 143.9	58.5 ± 15.3	22.8 ± 5.2	4.5 ± 1.7	1375.4 ± 117.4
1 d Abst + 1 d EE	7	171.2 ± 30.2	91.9 ± 10.4	2.5 ± 0.4	1800.1 ± 124.2	4.4 ± 0.6	2.1 ± 0.3	0.4 ± 0.4	192.6 ± 47.1
30 d Abst + 30 d EE	7	136.0 ± 11.2	81.8 ± 4.9	6.6 ± 3.2	2116.6 ± 171.2	30.0 ± 4.9	11.7 ± 1.6	4.4 ± 1.6	819.7 ± 125.9
30 d Abst + 1 d EE	9	132.8 ± 18.7	76.1 ± 6.9	3.6 ± 1.4	1696.4 ± 84.0	19.3 ± 2.6	8.7 ± 1.0	0.8 ± 0.1	392.7 ± 68.0

*indicates significant main effect of day;

^+^ indicates significant main effect of housing.

### 3.2 PNN intensity within the PFC increases following a cue-induced reinstatement test and EE

**Fig 2:**
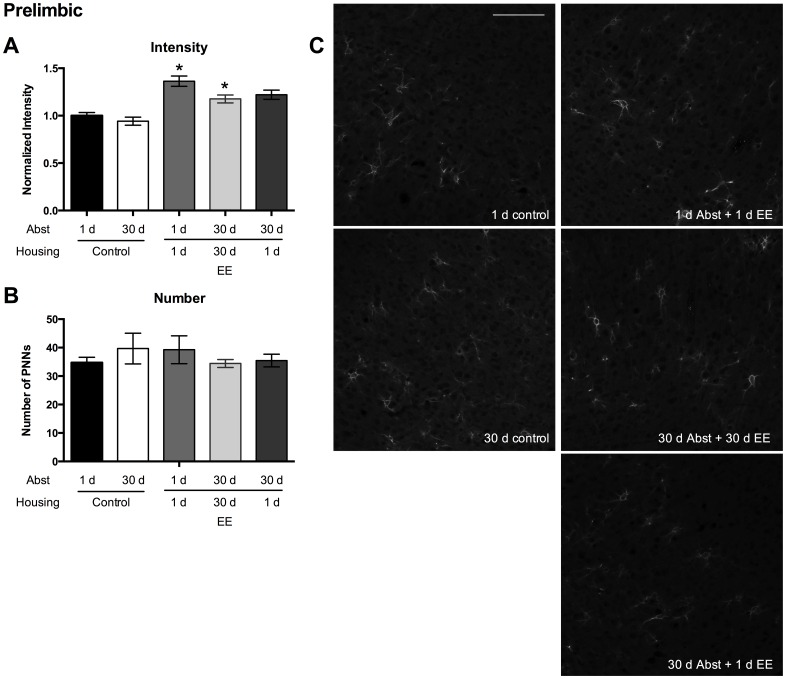
PL PNN intensities increase following EE and a cue-induced reinstatement test. (A) PNN intensity normalized to 1 d control housing. (B) The number of PNNs in the treatment groups. (C) Representative images from each group. n/group: 1 d control = 6; 30 d control = 4; 1 d Abst + 1 d EE = 7; 30 d Abst + 30 d EE = 7; 30 d Abst + 1 d EE = 9. Scale bar represents 100 μm. Data represent mean ± SEM. * p < 0.05 compared to 1 d control housing.

Within the PL region, intensity of individual PNNs increased following acute EE during acute abstinence or chronic EE compared to standard housing controls (Fig 2A). A Kruskal-Wallis test revealed a significant treatment effect (Fig 2A; p < 0.0001). PNNs from animals in the 1 d Abst + 1 d EE and 30 d Abst + 30 d EE groups displayed higher staining intensity than PNNs from animals in the control 1 d Abst group (Dunn’s multiple comparisons test, p’s < 0.05). PNNs from animals in the 30 d Abst + 1 d EE group did not display significantly higher staining intensity than PNNs from the control 1 d Abst group (Dunn’s multiple comparisons test, p = 0.17). These results suggest that exposure to EE during the entire length of abstinence prior to a cue-induced reinstatement of sucrose-seeking increases PNN intensity within the PL region. The number of PNNs did not differ among groups (Fig 2B; one-way ANOVA, p = 0.66).

**Fig 3:**
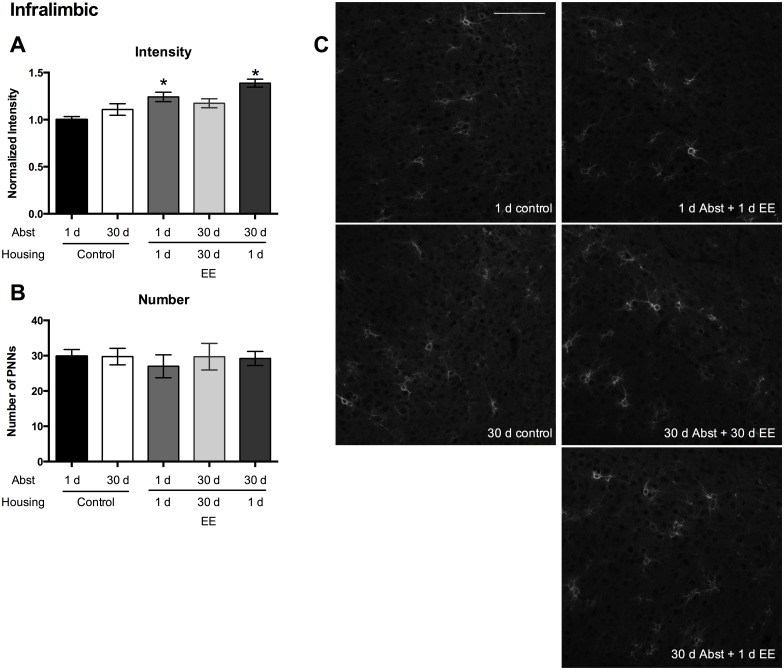
IL PNN intensities increase compared to the control (1 d Abst) group after a cue-induced reinstatement test. (A) PNN intensity normalized to 1 d control housing. (B) The number of PNNs in the treatment groups. (C) Representative images from each group. n/group: 1 d control = 6; 30 d control = 4; 1 d Abst + 1 d EE = 7; 30 d Abst + 30 d EE = 7; 30 d Abst + 1 d EE = 9. Scale bar represents 100 μm. Data represent mean ± SEM. * p < 0.05, compared to 1 d control housing.

Within the IL region, PNN intensity increased in the 1 day Abst + 1 d EE and the 30 d Abst + 1 d EE groups compared to the control 1 d Abst group (Fig 3A; Kruskal-Wallis test: p < 0.0001; Dunn’s multiple comparisons test: Control 1 d Abst vs. 1 d Abst + 1 d EE, control 1 d Abst vs. 30 d Abst + 1 d EE, both p’s < 0.01). The number of PNNs did not differ among groups (Fig 3B; one-way ANOVA, p = 0.95).

**Fig 4:**
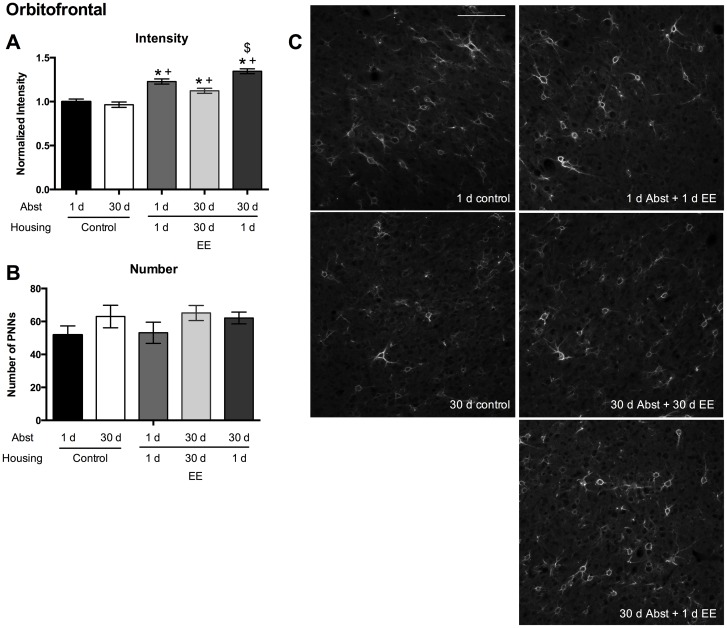
OF PNN intensities increase following acute EE and a cue-induced reinstatement test. (A) PNN intensity was normalized to 1 d control housing. (B) The number of PNNs in the treatment groups. (C) Representative images from each group. n/group: 1 d control = 6; 30 d control = 4; 1 d Abst + 1 d EE = 7; 30 d Abst + 30 d EE = 7; 30 d Abst + 1 d EE = 9. Scale bar represents 100 μm. Data represent mean ± SEM. * p < 0.05, compared to 1 d control housing; + p < 0.05, compared to 30 d control housing; $ p < 0.05, compared to 30 d Abst + 30 d EE.

PNN intensity increased within the OF region in all groups exposed to EE compared to standard control housing (Fig 4A; Kruskal-Wallis test, p < 0.0001; Dunn’s multiple comparisons test: Control 1 d Abst vs. 1 d Abst + 1 d EE, p < 0.0001; Control 1 d Abst vs. 30 d Abst + 1 d EE, p < 0.0001; Control 30 d Abst vs. 1 d Abst + 1 d EE, p < 0.0001; Control 30 d Abst vs. 30 d Abst + 30 d EE, p < 0.05; Control 30 d Abst vs. 30 d Abst + 1 d EE, p < 0.0001). Additionally, PNN intensity from animals receiving 30 d Abst + 1 d EE increased compared to staining from animals receiving 30 d Abst + 30 d EE (Dunn’s multiple comparisons test, p < 0.0001). These results suggest that any exposure to EE prior to a cue-induced reinstatement of sucrose-seeking increases PNN intensity within the OF region, while 1 d EE at the end of a 30 d Abst period further increases PNN intensity. The number of PNNs analyzed did not differ among groups (Fig 4B; one-way ANOVA, p = 0.28).

### 3.3 Novel acute EE alters PNN intensity in a region-dependent manner

**Fig 5:**
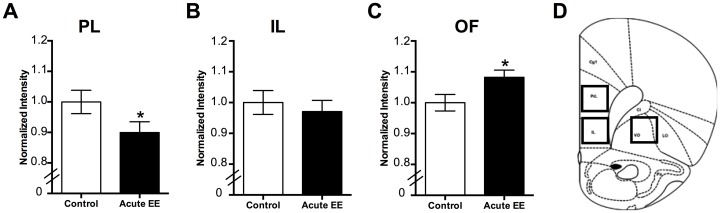
Exposure to 1 d EE changes PNN intensity within the PFC in a region-specific manner. (A) Intensity of PNNs within the PL region decreases following 1 d EE compared to standard housing controls. (B) Intensity of PNNs within the IL region do not change following 1 d EE compared to standard housing controls. (C) Intensity of PNNs within the OF region increases following 1 d EE compared to standard housing controls. (D) Representative region from the PFC (+3.2 from Bregma). Boxes indicate region included for PL, IL, and OF regions. Data represent mean ± SEM. * p < 0.05, compared to control.

Since we observed consistent and strong effects following acute EE, we examined the extent to which 22 hr of EE in sucrose-naïve rats altered PNNs within the PL, IL, and OF regions of the mPFC, which had not been previously measured (Fig 5D). Consistent with previous findings in other brain regions after longer EE [22–25], 1 d EE decreased the intensity of PNNs within the PL region (Fig 5A; Kolmogorov-Smirnov test, p < 0.05). 1 d EE did not alter intensity of PNNs within the IL region (Fig 5B, Kolmogorov-Smirnov test, p = 0.47). However, 1 d EE increased the intensity of PNNs within the OF region (Fig 5C; Kolmogorov-Smirnov test, p < 0.001). The number of PNNs did not differ between control and EE in any region (PL: control 31.17 ± 2.80, EE 36.00 ± 3.33, Students t-test, p = 0.29; IL: control 30.00 ± 3.58, EE 31.83 ± 1.05, p = 0.63; OF: control 64.17 ± 1.07, EE 64.33 ± 2.74, Student’s t-test, p = 0.97). These findings suggest that 1 d EE in naïve rats modifies PNN intensities in a region-specific manner.

## 4. Discussion

In the present study, we provide evidence that PNNs within the PFC are modulated in an experience- and region-specific manner. First, exposure to EE, either acute or chronic, during the entire length of abstinence following training of sucrose self-administration increases PNN intensity in the PL region following a cue-induced reinstatement session (Fig 2). Second, exposure to acute EE following training of sucrose self-administration increases PNN intensity in the IL region following a cue-induced reinstatement session (Fig 3). Third, exposure to any EE following self-administration training of sucrose increases PNN intensity in the OF region (Fig 4). Finally, in naïve rats that are not exposed to sucrose self-administration, exposure to acute EE slightly decreases the intensity of PNNs within the PL region, does not alter the intensity of PNNs within the IL region, and slightly increases the intensity of PNNs within the OF region (Fig 5).

### 4.1 Novel acute EE and PNNs

The present study is the first to show that 1 d EE without a history of sucrose self-administration decreases PNN intensity within the PL region and increases PNN intensity within the OF region. Our findings within the PL region are consistent with several previous studies showing a decrease in PNN intensity within the visual cortex, deep cerebellar nuclei, and somatosensory cortices following chronic EE [22–24]. However, our findings within the OF region are surprising in that PNN intensity *increases* following acute EE. This may be due to differences in the duration of EE. This study is also the first we are aware of to examine PNNs following a duration of only a single day of EE (1 d EE group), which is significantly less than most studies that examine 3 weeks to 1 month or longer of EE. This timeframe may enable us to observe a unique form of acute PNN plasticity. These differences may also be due to different functions of the brain regions examined. The PL region is involved in cognitive processing; whereas the OF is involved in complex sensory integration and value-based decision making [29–31].

The changes in PNN intensity after acute EE exposure may translate to altered output of mPFC neurons and changes in the local GABAergic network controlling mPFC output neurons [32]. Previous studies examining the effect of chronic EE on the visual system have reported decreased basal levels of GABA and increased levels of BDNF that accompany the decreased PNN intensities within the visual cortex [22, 23]. These studies suggest that EE modifies plasticity of the visual cortex by decreasing cortical inhibition and increasing plasticity mediators (including BDNF), which may then produce functional recovery of visual acuity following monocular deprivation.

### 4.2 Sucrose Self-administration, EE, and PNNs

Our study is also the first to report that in rats given sucrose self-administration training and EE (acute and/or chronic EE depending on the region) prior to a cue-induced reinstatement session, PNN intensity increases within the PL, IL, and OF regions of the PFC. One possible explanation is that EE alters the state of plasticity within the PFC so that subsequent cue reinstatement can influence changes in PNN intensity to a greater degree than that from rats held under standard housing conditions. In some instances, we found a significant relationship between brain region, cue-induced reinstatement, and treatment condition. For example, there was a positive and significant relationship between PNN intensity in the PL region (and in the OF region) and active lever presses on reinstatement day within the 30 d Abst + 1 d EE group (PL: R^2^ = 0.73, p < 0.005; OF: R^2^ = 0.46, p < 0.05). While these correlations are intriguing and point to a role for changes in PNNs in the effects of EE on cue-induced reinstatement, further research is needed to identify a causal relationship between PNNs and behavior in this model. Alternatively, EE may have increased PNN intensity independent of cue reinstatement such that reduced plasticity of the mPFC, as assessed by increased PNN intensity, renders the cue less salient and consequently reduces reinstatement.

Following 30 d EE and cue-induced reinstatement, Fos levels in the PL and OF regions are decreased, whereas following 1 d EE and cue-induced reinstatement, Fos levels within the IL region are increased [12]. The Fos findings are the reverse of our findings within the PL and OF: when we observed an increase in PNN intensity, there is a decrease in Fos levels [12]. However, within the IL region, our findings of increased PNN intensity match the previously reported increased levels of Fos following acute EE [12]. However, that study did not identify the phenotype of cells expressing c-Fos (GABAergic interneurons vs. glutamatergic pyramidal output neurons). Future studies are needed to identify the type of cell activated by cue and whether it is surrounded by PNNs.

Another possible explanation for the increase in PNN intensities is that exposure to 1 d EE creates a contrast in reward states for the animal. Given that 1 d EE increased PNN intensities in all regions examined, the novel experience of an enriched environment, which in our study included introduction to novel conspecifics and objects as well as more space [11], may permit the re-evaluation of reinforcement value of an experience—either EE or drug [12, 14]. In the classical “rat park” experiment, rats exposed to EE conditions chose to consume significantly lower levels of drugs than rats exposed to “deprived” conditions, which led to the idea that EE is valued as a greater reward than is a drug [33]. However, these changes in the relative value of a reward may be transient and depend on the current environment. The PNN intensities did not differ between 1 d or 30 d of standard housing, so although sucrose-seeking behavior incubates over abstinence, the PNNs do not change until EE is experienced.

We are only beginning to understand the consequences of changes in PNN intensity with regard to the influence of these changes on output of neuronal firing. PNN intensity correlates with the intensity of parvalbumin staining in GABAergic interneurons [34]. Initial stages of learning are associated with low parvalbumin staining, indicative of a reversion to an immature state with increased plasticity and a greater number of GABAergic synaptic inputs to these cells [35]. In contrast, strong memories are associated with increased parvalbumin staining intensity and a greater number of glutamatergic synaptic inputs. The EE-induced increase in PNN staining observed in the present study would suggest that there is elevated glutamatergic input to PNN-surrounded GABAergic interneurons, which could then increase GABAergic output to pyramidal neurons and potentially reduce cue-induced reinstatement. In addition, these fast-spiking GABAergic interneurons are critical for production of gamma oscillations, implicated in decision making, working memory, and impulse control [36]. Increased PNN intensity may indicate that EE suppresses gamma oscillations or disturbs the local excitatory/inhibitory balance in the mPFC to reduce cue-induced reinstatement.

## 5. Conclusions

The present study indicates that novel exposure to EE affects PNNs in the mPFC in a region-specific manner. Additionally, intensity of PNNs within the PL, IL, and OF regions of the PFC increase in response to EE followed by cue-induced reinstatement of sucrose. The increased PNN intensity is expected to have profound consequences for mPFC output to reward-related regions, such as the nucleus accumbens. Future studies will be needed to investigate the individual components of PNNs that underlie the changes in PNN intensity following sucrose self-administration, EE exposure, and cue-induced reinstatement.

## Abbreviations

Abst: abstinence
au: arbitrary unit
CPP: conditioned place preference
ECM: extracellular matrix
EE: environmental enrichment
IL: infralimbic
OF: orbitofrontal
PFA: paraformaldehyde
PFC: prefrontal cortex
PL: prelimbic
PNN: perineuronal net
WFA: *Wisteria floribunda* agglutinin
